# Emerging Bone Marrow Microenvironment-Driven Mechanisms of Drug Resistance in Acute Myeloid Leukemia: Tangle or Chance?

**DOI:** 10.3390/cancers13215319

**Published:** 2021-10-22

**Authors:** Marilena Ciciarello, Giulia Corradi, Dorian Forte, Michele Cavo, Antonio Curti

**Affiliations:** 1 Istituto di Ematologia “Seràgnoli”, Via Massarenti 9, 40138 Bologna, Italy; 2 Dipartimento di Medicina Specialistica, Diagnostica e Sperimentale, Università di Bologna, 40138 Bologna, Italy; giulia.corradi2@unibo.it (G.C.); dorian.forte2@unibo.it (D.F.); michele.cavo@unibo.it (M.C.); 3 IRCCS Azienda Ospedaliero-Universitaria di Bologna, Istituto di Ematologia “Seràgnoli”, 40138 Bologna, Italy; antonio.curti2@unibo.it

**Keywords:** hematology, chemotherapy resistance, bone marrow microenvironment, mesenchymal stromal cells, immune microenvironment

## Abstract

**Simple Summary:**

Despite high rates of remission obtained with conventional chemotherapy, the persistence of leukemic cells after treatments, eventually exiting in disease relapse, remains the main challenge in acute myeloid leukemia (AML). Increasing evidence indicates that, besides AML cell mutations, stromal and immune cells, as leukemic microenvironment components, may protect AML cells from therapies. Here, we will recapitulate emerging bone marrow (BM) microenvironment-dependent mechanisms of therapy resistance. The understanding of these processes will help find new drug combinations and conceive novel and more effective treatments.

**Abstract:**

Acute myeloid leukemia (AML) has been considered for a long time exclusively driven by critical mutations in hematopoietic stem cells. Recently, the contribution of further players, such as stromal and immune bone marrow (BM) microenvironment components, to AML onset and progression has been pointed out. In particular, mesenchymal stromal cells (MSCs) steadily remodel the leukemic niche, not only favoring leukemic cell growth and development but also tuning their responsiveness to treatments. The list of mechanisms driven by MSCs to promote a leukemia drug-resistant phenotype has progressively expanded. Moreover, the relative proportion and the activation status of immune cells in the BM leukemic microenvironment may vary by influencing their reactivity against leukemic cells. In that, the capacity of the stroma to re-program immune cells, thus promoting and/or hampering therapeutic efficacy, is emerging as a crucial aspect in AML biology, adding an extra layer of complexity. Current treatments for AML have mainly focused on eradicating leukemia cells, with little consideration for the leukemia-damaged BM niche. Increasing evidence on the contribution of stromal and immune cells in response to therapy underscores the need to hold the mutual interplay, which takes place in the BM. A careful dissection of these interactions will help provide novel applications for drugs already under experimentation and open a wide array of opportunities for new drug discovery.

## 1. Introduction

Acute myeloid leukemia (AML) originates from genetic or epigenetic lesions in hematopoietic stem cells (HSCs) or myeloid progenitors, which give rise to clonal expansion and accumulation of damaged undifferentiated cells in the bone marrow (BM) and peripheral blood (PB) [1].

Despite the high remission rates after induction therapy, treatment failure and relapse are still significant hurdles in AML. Relapse is mainly caused by tumor regrowth initiated by chemoresistant leukemic cells. Chemoresistant AML cells are thought to be enriched in leukemic stem cells (LSCs), an AML cell subpopulation, owing to quiescence and typically resistant to proliferation-targeting chemotherapy [2,3,4]. However, the precise characteristics of chemoresistant cells and the mechanisms underlying the switching between the drug-sensitive and -resistant state are still elusive, mainly in vivo.

AML chemoresistant cells are highly heterogeneous in vivo [5,6]. Chemotherapy resistance is thought to be controlled by intrinsic factors, i.e., growth factor signaling, epigenetic regulation, and metabolic reprogramming [7,8,9]. More recently, it has been outlined that immune and stromal cells could also promote drug resistance by influencing and modifying cell-intrinsic features [10,11]. In particular, among stromal cells, MSCs, initially recognized as essential components to the HSC niche, have revealed unexpected roles in leukemic cell protection from therapies.

Here, we will describe new emerging mechanisms driven by the BM microenvironment, emphasizing MSCs’ capacity to nurture a leukemia drug-resistant phenotype. We will also recapitulate the most recently acquired knowledge on the contribution of the immune system in the acquisition of resistance to therapy in AML. We will also speculate on the consequences of the stromal-immune BM microenvironment interplay on therapy response. Finally, we will discuss how these new pieces of the ‘chemoresistance puzzle’ can be exploited to design new combinatorial therapies and maximize treatment efficacy.

## 2. The Stromal-Dependent Anti-Apoptotic Effects 

**Figure 1:**
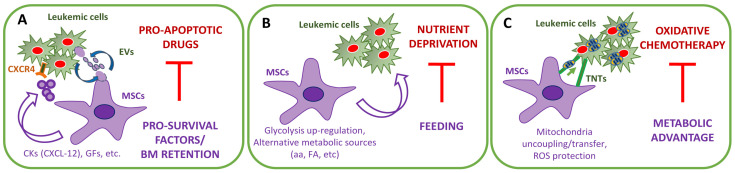
MSC-dependent mechanisms of resistance to therapy. Mesenchymal stromal cells (MSCs) support leukemic cells in diverse ways (purple): (**A**) supplying pro-survival factors (directly, mediated by extra-vesicle EVs or depending on BM retention), (**B**) providing metabolic substrates alternative to glucose, (**C**) rewiring metabolism. Each of these mechanisms interferes with a different therapeutic strategy (red). CKs, cytokines; GFs, growth factors; aa, amino acids; FA, Fatty acids; TNT, tunneling nanotubes; ROS, reactive oxygen species.

The final result of a successful cytotoxic treatment is the induction of programmed cell death, namely apoptosis. BM microenvironment offers protection against cytotoxic agents, activating anti-apoptotic signals and leading to enhanced cell survival and resistance to therapy (Figure 1A) [12,13]. In turn, the activation of signals which inhibit apoptosis is correlated with a poor response to chemotherapy in AML [14,15,16,17]. Soluble factors [18,19] and adhesion molecules [20] expressed by stromal cells activate pro-survival pathways. But whatever the upstream mechanism is, the activation of pro-survival pathways involves pro-/anti-apoptotic protein balance as a common target.

### 2.1. MSC-Dependent Pro-Survival Pathways

B-cell lymphoma 2 (BCL-2), a critical pro-survival factor, has an altered expression in leukemic cells [21] and is further up-regulated in co-cultures with stromal cells [19]. The highly selective BCL-2 inhibitor venetoclax (VEN) has proven highly effective in pre-clinical models [22,23]. However, its application as a single agent in clinical trials has been disappointing due to the development of resistance [24,25]. As a possible explanation, in vitro experiments have demonstrated that VEN efficacy was attenuated by cytokines (i.e., soluble factors) produced by stromal cells (Figure 1A) [26]. In particular, granulocyte-macrophage colony-stimulating factor (GM-CSF), and to some extent, granulocyte colony-stimulating factor (G-SCF), were shown to vicariate the effect of stromal cell-conditioned medium on AML cell viability, thus were indicated as the main culprits in stroma-mediated resistance to VEN [26]. Mechanistically, stromal cytokines activate Janus kinase (JAK)/STAT signaling and decrease the expression of BCL-2 relative to the other BCL-2 family members (i.e., BCLXL, MCL1, and BFL1). Consistently, drugs targeting anti-apoptotic proteins different from BCL-2 (e.g., navitoclax) maintain cytotoxic activity in the presence of the stroma [22,26]. Moreover, JAK-inhibitors synergize with VEN activity and override stroma protection [26].

New promising scenarios result from targeting induced myeloid leukemia cell differentiation protein (MCL-1), a BCL-2 family member essential for AML development and AML cell survival [27,28]. MCL-1 upregulation characterizes about half of resistant/relapsed AML patients and is associated with poor prognosis [29]. Specific inhibitors of MCL-1 and MCL-1-transcriptional regulator CDK9 have recently entered clinical trials [30,31,32]. Furthermore, to potentiate the anti-leukemic apoptotic mechanisms, the combination of MCL-1 and BCL-2 inhibition has been successfully applied in vitro and in vivo. MCL-1 inhibition sensitizes AML cells to anti-BCL-2 drugs (e.g., VEN), overcoming stroma-promoted resistance [32,33,34]. Interestingly, this process does not involve the dissociation of apoptotic/anti-apoptotic proteins. Indeed, MCL-1 has recently revealed unexpected functions beyond the cell-intrinsic anti-apoptotic effect [35]. MCL-1 promotes the adhesion-mediated interaction between AML cells and stromal cells. MCL-1 expression in AML cells positively correlates with the levels of Chemokine (C-X-C motif) receptor (CXCR) 4 and CD44, which are known to be involved in leukemia-MSC interactions, MSC-mediated support to AML cell survival, and engraftment [35]. Moreover, MCL-1 regulates redox and metabolic functions in AML cells. The MCL-1/BCL-2 combined inhibition prolongs the survival of mice-bearing xenografts derived from VEN-resistant patients, suggesting that these patients may potentially benefit from a similar combination. Although the underlying pathway remains unknown, the inhibition of CXCR4 and oxidative phosphorylation (OXPHOS) overcomes MSC-induced VEN resistance [35], suggesting that interference with stromal-mediated adhesion and metabolism regulation contributes to sensitizing AML cells to apoptosis induction.

Among factors regulating leukemia-MSC interactions, CXCL-12/CXCR4 axis plays a central role in leukemia resistance to therapies (Figure 1A) [36]. Stromal cell-derived factor (SDF)-1/CXCL-12, abundantly secreted by MSCs, stimulates malignant cell survival [37]. Moreover, despite both normal and malignant hematopoietic cells expressing CXCR4, the SDF-1 receptor, the expression level is higher in AML cells, having a major prognostic impact [38]. Leukemic cells utilize CXCR4 to enter niches normally restricted to HSCs, thereby competing for a microenvironment that favors their growth and survival. Disruption of CXCL-12/CXCR4 interactions with CXCR4 inhibitors efficiently blocks LSC homing to the BM niche and likely sensitizes leukemic cells to chemotherapy (Figure 1A) [39,40]. Based on their ability to mobilize leukemia cells out of protective BM niches, CXCR4 antagonists have been explored as therapy in combination with cytotoxic drugs, including VEN [36,41].

### 2.2. The Emerging Role of Extracellular Vesicles

Among the extrinsic microenvironmental factors with a putative relevance in AML chemoresistance, extracellular vesicles (EVs), including exosomes, ectosome, and microvesicles, have progressively attracted crucial attention (Figure 1A) [42]. Several types of cells, including immune and stromal cells, under physiological and pathological conditions, release EVs containing tumor-derived material (DNA, RNA, proteins, and lipids) and act as a reservoir of clinically relevant biomarkers, and as carriers of complex intercellular information within the microenvironment, even in AML [42]. In AML patients, EV levels are elevated in plasma at diagnosis and remain elevated in complete remission (CR) after chemotherapy [43]. Additionally, the miRNA content of EVs can predict both the risk of relapse and death in AML patients [44]. Accumulating evidence has reported a key role for EVs in drug resistance and immune response modulation [45,46].

Interestingly, EVs from healthy or leukemic niches reveal differences in chemoprotection. The EVs from AML-MSCs (AML-MSC-EVs) protect AML cells against cytarabine or AC220 (FLT3 kinase suppressor) and contain higher levels of well-known clinical risk factors, such as transforming growth factor-β1 (TGF-β1) and miR-155, compared to EVs from healthy donors [47]. Consistently, AML-MSC-EVs regulate the AML progression promoting cell proliferation, migration, and invasion, inhibiting GSK3β expression and activating Wnt/β-catenin signaling in AML cells [48].

By contrast, EVs derived from healthy MSCs (HD-MSC-EVs) suppress cell proliferation and promote apoptosis in KG-1a cells, likely acting through miR-124-5p [49]. HD-MSC-EVs transfer miR-222-3p to THP-1 cells and exert pro-apoptotic effects targeting the IRF2/INPP4B signaling pathway [50]. However, in another study HD-MSC-EVs promote resistance to cytarabine via the upregulation of S100A4, a calcium-binding protein with a prominent role in tumorigenesis [51]. Different outcomes may be due to the EV population heterogeneity, the contained cargo, and the cell line used for the study.

In addition, AML cells generate EVs (AML-EVs). EV transfer from AML cells to MSCs might remodel the BM cell composition and concomitantly influence AML survival [52]. AML-EVs transfer to MSCs, BMP-2, a protein upregulated in AML and related to endoplasmic reticulum (ER) stress. BMP-2-transfer activates the unfolded protein response pathway (UPR) as an enhancing extrinsic mechanism of chemoresistance [53]. Interestingly, AML-EVs upregulate IL-8 in MSCs, promoting AML cell resistance to etoposide [45].

Intriguingly, blocking the effects of EVs in the cross-talk between leukemic cells and the supportive microenvironment might be considered a promising therapeutic approach [54]. To the best of our knowledge, few data are explicitly reported in AML. However, Paggetti et al., using a heparan sulfate analog, suggest a way to counteract tumor growth, decreasing the uptake of chronic lymphocytic leukemia (CLL)-derived EVs by target cells [55]. Accordingly, in lymphoma cell lines, blocking EV release through indomethacin decreases tumor progression [56]. To date, despite the fascinating involvement of EVs in AML resistance, the standardization of protocols for EV isolation and the effectiveness and safety of blocking EV cargo need to be addressed before exploiting EV targeting as therapy.

## 3. The Stromal-Dependent Metabolic Reprogramming

Similar to other cancer cells, AML cells hold the capacity to adapt their metabolism to microenvironment conditions and stimuli [57]. AML cells put in the field different strategies to sustain tumoral accelerated metabolism and adequately feed the tricarboxylic acid (TCA) cycle. Enhanced glucose catabolism mainly provides advantages, such as rapid adenosine triphosphate (ATP) production and building blocks for nucleotides, amino acids, and lipids [58]. In contrast, metabolic source alternatives to glucose, such as amino acids [59] and fatty acids (FA) [60], operate as a supplier of the TCA cycle for energy production [57]. Metabolism rewiring confers to AML cells a selective advantage over normal HSCs and protection from therapeutic agents (Figure 1B,C) [57]. At the same time, the re-organization of metabolic pathways frequently renders AML cells autotrophs for some nutrients offering therapeutic opportunities.

### 3.1. Glucose Addiction

AML samples show a higher glycolytic flux compared to healthy controls [61]. The glycolytic rate divides AML patients into subgroups with different prognostic values [62]. Altered glucose metabolism is also associated with resistance [63]. Blocking glycolysis has been revealed as a promising strategy against AML cells [64,65]. The stroma upregulates glycolysis in AML cells through a not completely defined mechanism [66], but depending, at least in part, on mammalian Target of Rapamycin (mTOR) kinase hyper-activation. Stromal interaction increases the glycolytic flux of AML cells through the CXCR4/CXCL12 axis, which activates mTOR [67]. Stromal contact also upregulates the AML cell expression of Glucose transporters (GLUT1-4), paralleled by an increased glucose uptake [67]. Targeting glycolytic pathways overrides stroma-mediated protection [67]. mTOR inhibition by rapamycin decreases glucose uptake and downregulates glycolytic pathway genes in AML cell lines [68,69]. Although with conflicting results, rapamycin has been observed to cross-react with glucose deprivation or glycolysis inhibiting drugs [64,69]. Moreover, a highly selective mTORC1/2 inhibitor is able to target leukemic cells within the BM microenvironment [70]. Thus, stroma-mediated ‘glucose addiction’, on the one hand, increases the resilience of AML cells to cytotoxic agents (Figure 1B) [13,67], but on the other hand, can be exploited in novel drug combinations.

### 3.2. Biofuels Alternative to Glucose: Glutamine, Asparagine, and Cysteine

Unlike normal HSCs, leukemic cells are highly dependent for survival and proliferation on asparagine (ASN) and glutamine (GLN) as alternative carbon sources [71]. The inhibition of the synthesis or utilization of GLN and ASN has detrimental effects on AML cells. Targeting such metabolic vulnerability, e.g., amino acid deprivation, can be considered a successful strategy to target AML cells. However, it must be taken into account that stromal cells in the leukemia BM microenvironment, particularly MSCs and adipocytes, can provide AML cells with different metabolic substrates other than glucose (Figure 1B). Specifically, MSCs show a strong overflow metabolism that leads to the secretion of diverse amino acids that can be exploited by AML cells as metabolic substrates [72,73]. 

Inhibitors of Glutaminase (GLS), the enzyme deaminating GLN to the alpha-ketoglutarate (α-KG) precursor glutamate (GLM), reduce AML cell growth and induce apoptosis [71,74]. IDH1 and IDH2 mutants, which cannot produce α-KG through alternative pathways, are more susceptible to GLS inhibition [75].

The protective effect of stromal cells on the apoptosis induced in AML cells by GLS inhibition has not been investigated yet. However, it is known that the release of GLN by adipocytes causes leukemia cell resistance to Asparaginase (ASNase) [76]. ASNase is a universally used component of acute lymphoid leukemia (ALL) treatment and in the context of multiagent chemotherapy. Leukemia cells adapt to ASNase treatment by increasing the synthesis and transport of GLN [76]. Thus, while considering detrimental side effects [77], targeting GLN metabolism is necessary to override ASNase resistance [78]. 

In a murine model of AML, it has been demonstrated that aspartate produced by MSCs, fuels pyrimidine synthesis in leukemic blasts. Thus, amino acid production by MSCs could also mediate resistance to nucleotide deprivation-based chemotherapy (Figure 1B) [77].

To sustain their energy metabolism, AML cells, specifically LSCs, also rely on cysteine [79,80]. MSCs, but not leukemic cells, import cystine and convert it to cysteine. In turn, cysteine is secreted by MSCs and uploaded by leukemic cells to generate glutathione (GSH) [81,82]. Thus, stromal cells regulate cysteine metabolism and increase GSH levels enhancing leukemia cell survival and protecting them from drug-induced cytotoxicity and oxidative stress (Figure 1C) [81]. As discussed below, targeting GSH metabolism has been considered a promising avenue to eradicate AML [74,83].

### 3.3. Biofuels Alternative to Glucose: Fatty Acids

Besides glucose and amino acids, fatty acids (FA) can also be utilized by AML cells as alternative sources of energy [7] and converted in acetyl-CoA to feed the Krebs cycle and OXPHOS aimed at ATP production [84]. Tumor cells obtain FA from their microenvironment through lipolysis of triglycerides stored in the adipocytes. Lipolysis is initiated by activating the β-adrenergic receptor, which stimulates lipolytic enzymes, e.g., the hormone-sensitive lipase (HSL) [85]. The ‘FA store’ is particularly rich in AML. BM stromal cells are reduced in AML [86,87], but adipocytes are abundant and increase in elderly patients [88,89]. Tabe et al. [60] have recently discussed the mechanisms by which the stromal BM microenvironment and specifically adipocytes support leukemic cell survival and metabolic demand of FA, as well as the therapeutic potential of fatty acid oxidation (FAO) inhibitors. Here, we will briefly recapitulate some key concepts.

- Leukemia cells prompt their microenvironment to increase FA production and availability.

In the BM microenvironment, adipocytes induce a pro-inflammatory/secreting cytokine-phenotype in leukemic cells, which causes increased lipolysis in adipocytes [90]. AML cells also directly promote lipolysis by inducing in the adipocytes the phosphorylation and activation of HSL [91]. Increased lipolysis in adipocytes results in a greater FA release [90,91].

- FAO promotes AML cell survival.

The FA released by adipocytes are captured by AML cells with the help of two fundamental players: the fatty acid transporter CD36 and the chaperon fatty acid-binding protein 4 (FABP4) [91,92]. FABP4 transports FA out of the adipocytes and in AML cells to the mitochondria, where FA are oxidation substrates. FA, transferred in the nucleus, bind to Peroxisome proliferator- activated receptor (PPAR)-γ and induce the transcription of downstream targets, including CD36 and FABP4, and the anti-apoptotic protein BCL2. Disruption of FAO in BM adipocytes reduces their protective effect [93]. FABP4 inactivation in the adipocytes has a significant inhibitory effect on co-cultured AML cells. Moreover, FABP4 inactivation in AML cells prolongs survival in a murine leukemia model [91]. By inducing lipolysis in adipocytes, a CD36^+^ LSC subpopulation holds an increased FA uptake and oxidation rate, facilitating their survival [90]. Additionally, MSCs favor FAO in AML cells and activate the anti-apoptotic machinery [93]. However, adipocytes seem to be more effective if compared with MSCs. Adipogenic differentiation of MSCs was associated with enhanced leukemia engraftment in a mouse model [94].

- BM-adipocytes impair the efficacy of anti-leukemia drugs.

‘FA addiction’ of AML cells can be exploited for therapy purposes. Pharmacological inhibition of FAO effectively sensitizes human AML cells to apoptosis induction [93]. However, the adipocyte pro-survival effect can interfere with this kind of therapy (Figure 1B). Co-culture with BM-derived adipocytes inhibits the anti-leukemic effect of the FAO inhibitor, avocatin B [95]. Accordingly, in vivo, the relapse rate was higher in obese mice than their normal-weight counterparts [93,96].

FAO inhibition can trigger metabolic adaptation. FAO inhibition causes adaptive stimulation of FABP4-mediated FA uptake, enhances glucose uptake and metabolic switching to glycolysis [95]. Combinatorial regimens can override microenvironment-dependent metabolic adaptation. FAO inhibitors are highly synergistic with conventional treatment, like paclitaxel or cytarabine [95,97].

### 3.4. Glycolysis versus OXPHOS

Recent findings indicate that, unlike other cancer cells, in AML cells, the Krebs cycle is intact, and OXPHOS is highly active [23,93]. In particular, LSCs considered the real perpetrators for the propagation of AML rely on OXPHOS to generate ATP [98]. Thus, chemo-resistant AML cells are not simply enriched in LSCs, as previously thought, but own the specific feature to rest on mitochondrial OXPHOS for their high metabolic demand [7,23,99,100]. In particular, AML cells hold a distinctive high OXPHOS gene signature that is also predictive for treatment response in a xenograft model [7]. Moreover, modulation of mitochondrial OXPHOS status markedly affects chemotherapy efficacy in vitro and in vivo [7].

However, MSCs can induce a ‘Warburg phenotype’ in AML cells (i.e., aerobic glycolysis for energy metabolism) where the oxidative capacity impairment is mimed by mitochondrial uncoupling (i.e., the abrogation of ATP synthesis in response to mitochondrial potential). MSCs, increase leukemia cells’ expression of uncoupling protein 2 (UCP2), a mitochondrial inner membrane protein that dissipates the electrochemical gradient generated by the mitochondrial respiration chain. Uncoupled mitochondria are more resistant to cytotoxic insults, produce less reactive oxygen species (ROS), and block the activation of the intrinsic apoptotic pathway (Figure 1C) [66,101]. 

Acquiring metabolites and organelles, like mitochondria, is a cancer cell alternative strategy to building up ATP production. AML cells present a higher number of mitochondria in comparison with normal HSCs [102]. MSCs could provide additional mitochondria to AML cells through a process still to be defined but which likely involves tunneling nanotubes (TNTs) and/or endocytosis and requires a cell-to-cell contact [103,104]. AML cells, through the nicotinamide adenine dinucleotide phosphate (NADPH) oxidase-(NOX)-2 activity, locally increase oxidative stress and boost MSC mitochondria production [103]. The extra-mitochondria are transferred to AML cells by activating peroxisome proliferator-activated receptor γ coactivator (PGC)-1α, the master regulator of mitochondrial biogenesis [105]. Mitochondrial transfer increases upon chemotherapy and is proposed as an additional resistance mechanism by reducing mitochondrial depolarization and chemotherapy-induced oxidative stress (Figure 1C) [104]. Moreover, AML cells equipped with extra-mitochondria could acquire other anti-apoptotic proteins, e.g., from the BCL-2 family, gaining a survival advantage and resistance to standard therapy, as mentioned before [106]. Thus, targeting MSC-mediated mitochondria transfer, specifically NOX2 and PGC-1α, could emerge as an intriguing therapeutic approach, especially as normal CD34^+^ HSCs are less prone to receive extra-mitochondria, giving a chance for targeted therapy with limited detrimental effects [104].

More generally, mitochondrial OXPHOS represents a putative point of vulnerability for AML chemo-resistant cells, including LSCs. The inhibition of mitochondrial metabolism selectively targets LSCs, and high OXPHOS AML cells that are metabolically inflexible, i.e., cannot shift to glycolysis when mitochondrial respiration is inhibited [23]. Initial results suggest that targeting mitochondrial OXPHOS may be a promising strategy in AML therapy alone or in combination with chemotherapy [23,107]. Recently, a highly potent and selective small-molecule inhibitor of complex I of the mitochondrial electron transport chain (IACS-010759) has shown promising anti-leukemic activity. In glycolysis-deficient and OXPHOS-reliant AML cells, OXPHOS disruption creates a microenvironment of energy and macromolecule depletion, impairs nucleotide biosynthesis, leading to AML cell cycle arrest and apoptosis [108]. Combining VEN with hypomethylating agents is proposed to selectively target amino acid dependent OXPHOS in LSCs [79,99]. Consistently, VEN and hypomethylating agent synergistic activity have also been demonstrated in vivo in AML patients [99,109]. MSCs could negatively affect AML cell resistance to OXPHOS-targeting drugs by increasing the bioenergetic capacity of AML cells [110]. Thus, in this case, MSC-protective contribution must be considered (Figure 1C). 

### 3.5. ROS and Redox Metabolism

Besides generating metabolic intermediates, AML cells utilize alternative energy sources to counteract oxidative stress, a metabolic state characterized by damage to DNA, amino acids, lipids, and carbohydrates [52]. Oxidative stress is due to a defect in antioxidant defense or excessive ROS production. ROS are highly active molecules generated by OXPHOS in the mitochondria or partly by the NOX complexes’ activity [111].

Physiologically, ROS participate in the crosstalk between HSCs and their BM microenvironment, including MSCs, affecting the maintenance of stem cell functions, differentiation, and migration [112]. The release of ROS within the BM microenvironment may represent a mechanism of apoptosis induction. Thus, modulation of ROS levels can be exploited as a therapeutic strategy. Intuitively, chemotherapy efficacy may lie on increased ROS-mediated cellular damage [113]. However, multiple genes can potentially contribute to ROS production, and it might be worthy of avoiding compensatory effects dangerous for the patients. Moreover, HSCs are sensitive to ROS, hence therapeutic intervention might impair normal hematopoiesis. Finally, ROS signaling could increase genomic instability and favor the survival of resistant cells to therapy, promoting, in turn, disease progression [114]. 

Excellent reviews reported different approaches to modify ROS levels with an anti-leukemia purpose: lowering the ROS level to prevent their role in cellular transformation or counteracting the redox adaptation by inducing oxidative stress with elevated ROS levels [115,116].

However, to date, not much attention has been given to microenvironmental driven mechanisms of resistance depending on ROS. MSCs provide AML cells protection against excessive ROS levels (Figure 1C). In vitro, MSCs supply a chemo-resistant niche for leukemic blasts promoting a quiescent phenotype in a side population with a low ROS transcriptional pathway [117]. In leukemic cells, the presence of BM-MSCs promotes the overexpression of GPX3, the cytoplasmic relocalization of Nrf2, and inactivation of p38MAPK with a concomitantly decrease in ROS levels. Of note, a reverse effect was observed in BM-MSCs in contact with leukemic cells, with a particular rise in ROS levels, decrease in GPX3 expression, and nuclear relocalization of Nrf2 [118]. One explanation could be ascribable to a mechanism of ROS transfer between AML cells and MSCs mediated by Connexin(CX)-43, a gap junction protein [119]. Remarkably, CXs are involved in the transfer of ROS from HSCs to the BM microenvironment. Disruption of gap junctions attenuates AML chemoresistance induced by MSCs, suggesting CX-mediated interactions between AML cells and their BM microenvironment as a putative therapeutic target for AML [119].

As mentioned before, chemoresistance mechanisms involving ROS modulation might be associated with metabolic rewiring induced by the BM microenvironment [78]. BM-MSCs (i.e., nestin^+^ cells) increased the bioenergetic capacity of AML cells contributing to their chemoresistance (Figure 1C) [110]. In particular, MSCs increase the energy production of LSCs, promoting GSH-mediated antioxidant defense against excessive ROS. AML cells can instruct MSCs to supply protection against ROS. Of note, mitochondria are the main source of ROS, which are key regulators of mitochondrial transfer in AML [120]. The co-culture with AML blasts increases ROS levels and oxidative stress in MSCs, leading to the transfer of mitochondria from MSCs to AML cells, feeding the mechanism of resistance to chemotherapy mentioned before [103]. Thus, the MSC-mediated support allows AML cells to satisfy their high metabolic demands and resist chemotherapy-induced oxidation (Figure 1C) [110].

Microenvironmental metabolic reprogramming linked to redox metabolism could offer a complementary therapeutic strategy to target AML cells. The synergistic effects of FAO inhibitors and cytarabine cause ROS induction and apoptosis in AML cells abrogating the BM adipocytes-induced AML protective effects. Specifically, combination treatment increases ROS production only in AML cells co-cultured with adipocytes compared to mono-cultured cells [95]. We also reported a synergistic effect of GSH inhibition using buthionine sulfoximine (BSO) with standard chemotherapy. In particular, the adjuvant GSH inhibition improved survival rate and significantly delayed leukemia relapse in mice. Thus, targeting the antioxidant support mediated by nestin+ MSCs could be considered a further therapeutic option [110].

## 4. The Immune BM Microenvironment in Chemoresistance

A functional immune system is required to prevent cancer onset and progression. Innate and adaptive immune cells recognize and destroy tumor cells in a process collectively indicated as cancer immune surveillance [121,122]. However, in parallel, cancer cells avoid the immune response exploiting many strategies of immune escape [123].

### 4.1. AML-Mediated Mechanism of Immune Evasion

**Figure 2:**
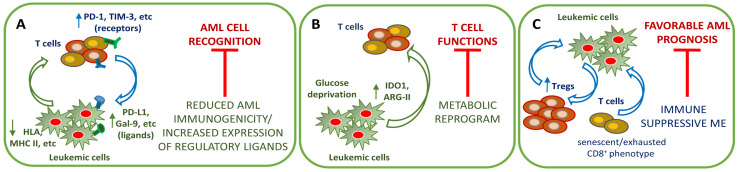
Interactions between AML cells and T cells potentially mediate therapy resistance. (**A**) Intrinsic immune evasion mechanisms adopted by AML cells to escape immune system recognition and destruction (green). These mechanisms include the reduction of AML immunogenicity (e.g., genomic loss of HLA haplotype, downregulation of MHC class II gene expression) and expression of regulatory ligands, such as PD-L1 and Gal-9 on AML cells interacting with their IRs on immune cells (PD-1, TIM-3). (**B**) AML cell aberrant metabolism, through the expression of metabolic enzymes (e.g., ARG-II and IDO1), leads to glucose deprivation, amino acid starvation, accumulation of kynurenines, etc., affecting T cell functions. (**C**) The immunologic status of AML patients at diagnosis, including an increased number of Tregs in PB and BM and an exhausted and senescent phenotype of CD8^+^ cells, has been associated with poor prognosis in AML patients. HLA, human leukocyte antigens; MHC, major histocompatibility complex; PD-L1, programmed death-ligand 1; Gal-9, Galectin-9; PD-1, programmed cell death protein 1, TIM-3, T cell immunoglobulin and mucin domain 3; ARG-II, arginase; IDO1, indoleamine 2,3 dioxygenase; ME, microenvironment.

Immune escape in AML includes AML cell-intrinsic mechanisms to reduce their immunogenicity, such as the low expression of AML tumor antigens, the genomic loss of human leukocyte antigens (HLA) haplotype [124], the downregulation of major histocompatibility complex (MHC) class II gene expression [125], and the defective formation of immune synapses (Figure 2A) [126]. Moreover, AML cells cooperate to create a suppressive microenvironment, exploiting physiological mechanisms of immune tolerance [127]. The interaction between the immune checkpoint (IC) receptors and their ligands preventing the proliferation and activation of immune cells, which regulate autoimmunity and self-tolerance, is a well-known immune escape mechanism in AML [128]. T cells express the respective co-inhibitory receptors (IRs), including programmed cell death protein 1 (PD-1), cytotoxic T-lymphocyte associated protein 4 (CTLA-4), T cell immunoglobulin and mucin domain 3 (TIM-3), lymphocyte activating-3 (LAG-3), and T cell immuno-receptor with immunoglobulin and immunoreceptor tyrosine-based inhibitory motif domains (TIGIT) [128]. At the same time, AML cells overexpress the regulatory ligands, such as programmed death-ligand 1 (PD-L1), B7-H3 (CD276), and Galectin 9 (Gal-9), CD112, CD155 (Figure 2A) [129,130]. These mechanisms of AML immune evasion are recognized as fundamental for the relapse after allogeneic hematopoietic stem cell transplantation (allo-HSCT).

AML cells can also favor an immunosuppressive niche by directly promoting T regulatory cell (Treg) induction (Figure 2C) [131,132] or through diverse indirect mechanisms: secretion of soluble factors altering T-cell immune responses (i.e., IL-10, TGF-β) [133], the release of EVs with immunosuppressive functions [134,135] or exploitation of the activation of other immunosuppressive cells, i.e., MSCs, M-2 polarized macrophages [136], and myeloid-derived suppressor cells [137].

Last but not least, the AML cell aberrant metabolism mentioned above can act as a checkpoint limiting immune-mediated leukemic cell destruction. AML cells express metabolism-regulating enzymes with an immunosuppressive function, such as Indoleamine 2,3-Dioxygenase 1 (IDO1) and Arginase II. In particular, Arginase II leads to arginine deprivation, which causes T cell apoptosis and autophagy [138]. IDO1 catalyzes the first and rate-limiting step of tryptophan degradation along the kynurenine pathway [139]. IDO1 activity increases T cell apoptosis, reduces T cell proliferation, and induces Tregs (Figure 2B) [140,141]. IDO1 expression in AML cells has been correlated to poor clinical outcomes [142,143]. 

Glucose depletion in the BM microenvironment operated by high consuming AML cells could impact immune cells’ survival, proliferation, and functions (Figure 2B). Overall, metabolic remodeling of the BM microenvironment by AML cells favors the induction and the survival of immune-regulatory cells, such as Tregs [144].

### 4.2. Immune System Dysregulation

Besides AML cell-directed immune evasion, immune system dysregulation, leading to impaired recognition of altered cells and hampered anti-tumor immune response, has been extensively described in AML patients. Comparing the immune profile changes among the different leukemias and healthy subjects suggests a disease-related immune-regulation [145,146]. Immune cell abnormalities in AML include proliferation and activation status, misproportion between T effectors (Teffs) and Tregs in favor of the latter, CD8^+^ T cell exhaustion and senescence [147], increased suppressive macrophages [136], and myeloid-derived suppressor cells (MDSCs), increased release of immunosuppressive factors [123,148], defective antigen presentation by antigen presenting cells (APCs) [123], suppression of natural killer (NK) cell-mediated cytotoxicity [149,150]. In particular, a specific enrichment in T cells expressing PD-1, a marker of exhaustion [151], has been identified in leukemias, including AML [152]. Furthermore, in AML, specific immune evasion mechanisms can occur in the BM, which is characterized, differing from the PB counterpart, by a decreased lymphocyte population due to blast expansion [152].

A comprehensive discussion of all these mechanisms is out of the scope of the present review [128,130]. Below we will focus on Tregs and Teffs. Tregs are a specialized suppressive subset of CD4^+^ T cells showing a high expression of CD25 (interleukin [IL]-2 receptor) and transcription factor FoxP3 [153]. Tregs are fundamental to control inflammatory status and establish peripheral tolerance [154]. The immunosuppressive effect of Treg is necessary to prevent and minimize graft versus host disease (GVHD) after allo-HSCT. Thus, the presence of Tregs can have a beneficial impact on AML patients after transplantation [155].

Besides their physiological role, Tregs are also involved in cancer immune evasion and progression [156,157]. Thus, Tregs act as a ‘double-edged sword’: on the one hand, they protect the host from autoimmunity, on the other hand, they can be exploited by cancer cells to hamper antitumor immunity. Tregs increase among tumor-infiltrating lymphocytes is associated with poor survival in many solid cancers [158]. Although Tregs’ role in pathogenesis, prognosis, and treatment of hematological malignancies is still under debate [159], a higher number of Tregs is associated with adverse outcomes in patients [160,161,162]. In particular, an increased frequency of Tregs associated with a hyper-activated and suppressive phenotype has been identified in BM and PB of AML patients (Figure 2C) [163,164]. Moreover, AML harbors an immune suppressive microenvironment associated with poor prognosis [165,166].

Our unpublished data demonstrate that in the BM of a subgroup of AML patients at diagnosis, the percentage of Tregs is higher compared to healthy donors. Interestingly, AML patients with lower PB Treg frequency at diagnosis respond better to induction chemotherapy [164]. Conversely, elevated PB Treg frequency, persisting after CR, is correlated to an increased risk of relapse [165,167]. Murine models of AML confirm an increased presence of Tregs in the liver and spleen, paralleled by AML cell engraftment, and a correlation between Treg infiltration and leukemia progression [168]. 

Treg reduction enhances an anti-leukemic effect mediated by CD8^+^ cells and delays disease progression [169]. Thus, temporary removal of Tregs is able to evoke and enhance antitumor effects resulting in suppression of tumor outgrowth [170,171]. Moreover, Treg depletion, by using an interleukin-2 diphtheria toxin, improved the long-term survival of mice [168]. However, the antitumor effect can be associated with autoimmune reactions, a side effect that must be considered in the immunotherapy protocols. 

The mechanisms underlying the abnormal Treg increase in AML are still under investigation; migration, local expansion, and conversion from conventional CD4^+^ T cells may be involved [169,172,173]. Treg accumulation can be reduced by blocking CCL3-CCR1/CCR5 and CXCL12-CXCR4 axes, suggesting increased migration mechanisms [169].

Other T cell dysfunctions have been associated with the response to induction chemotherapy in AML patients. In the early ‘70s’ a correlation between a diminished function of T helper cells and the chemotherapy response and maintenance of remission was suggested [174,175]. An altered CD8^+^ T cell function, characterized by features of exhaustion and senescence, has been described in the PB of AML patients at diagnosis (Figure 2C) [147]. Furthermore, the phenotypic and transcriptional profile of CD8^+^ T cells is different in patients achieving CR after chemotherapy compared with those who did not achieve CR. After therapy, the gene expression profile of CD8^+^ T cells is similar to that of healthy donors, indicating that the reversion toward a healthy-like pattern of gene expression in CD8^+^ T cells is essential to achieve CR. The restoration of T cell functions, namely the downregulation of apoptotic and exhaustion markers and the upregulation of co-stimulatory pathways in the BM CD8^+^ T cells, correlates with an effective therapeutic response [147]. Interestingly, AML blasts can directly alter CD8^+^ T cell expansion, viability, and expression of senescence and co-signaling markers, including the IRs, PD-1, 2B4, and CTLA-4, and the co-stimulatory receptors, ICOS and OX40 [147]. 

Besides a senescent and exhausted phenotype, the expression of PD-1 on CD8^+^ T cells is also predictive of poor overall survival and event-free survival in AML patients [176]. In addition, in patients with de novo AML, the increased co-expression of PD-1 and TIGIT on CD8^+^ T cells is associated with the failure to achieve remission after induction chemotherapy [177]. CD8^+^ T cell dysfunction may be revealed as critical prognostic biomarkers and help understand the most efficient therapeutic strategies to improve the clinical prognosis of AML patients.

Of note, a growing body of evidence indicates that the anti-tumor effect of some drug regimens is mediated at least in part by immune activation. Thus, the AML cell-dependent immune microenvironment modulation, the increased frequency of Tregs, and the unbalancing of Treg/Teff ratio all contribute to creating an immune-suppressive microenvironment in AML that can result in the inhibition of immune-mediated anti-tumor effects and response to therapy.

### 4.3. Immune System Modulation as a Chance for Therapy 

**Figure 3:**
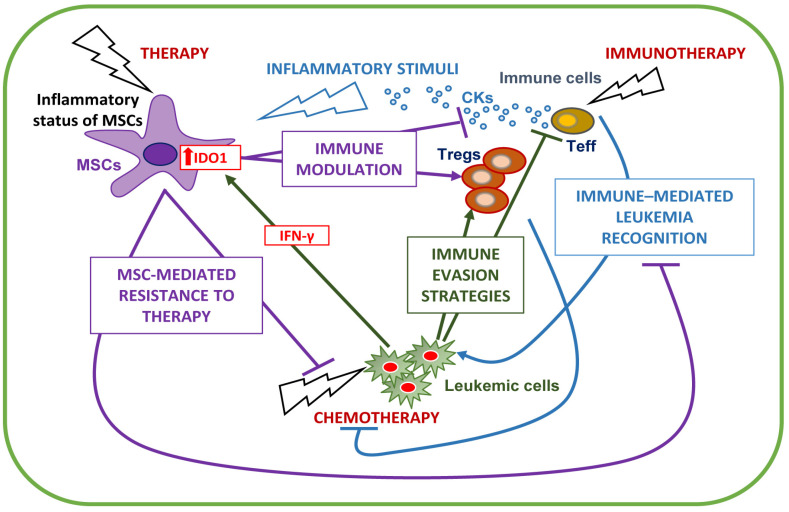
MSC, immune, and leukemic cell mutual interactions. Mesenchymal stromal cells (MSCs) mediate drug resistance through the mechanisms described in Figure 1 (purple). The therapy can influence MSC inflammatory status, modulating MSC immune suppression activity, which leads to suppression of T cell function and induction of Tregs (purple). Immune cell functions are also affected by leukemic cells, which favor an immune-suppressive environment to promote immune escape, through several cell-intrinsic (see Figure 2A,B) and cell-extrinsic mechanisms (i.e., EV release, aberrant metabolism, and the exploitation of other cells through CKs, e.g., IFN-γ which leads to IDO1 upregulation in MSCs) (green). A dysfunctional immune system can also influence the response to therapy (blue) (see Figure 2C). On the contrary, immunotherapy harnesses immune cells to recognize and destroy leukemic cells (blue). At the same time, the activation of the immune system is paralleled by an inflammatory status that could reprogram the MSC behavior, tuning immune suppression (purple). Tregs, T regulatory cells; T eff, T effector cells; EVs, extracellular vesicles; CKs, cytokines; IFN-γ, interferon-γ; IDO1, indoleamine 2,3, dioxygenase 1.

On the other side of the coin, the immune system can be successfully harnessed to eliminate leukemia cells (Figure 3). After chemotherapy, higher recovery of lymphocytes and neutrophils is associated with increased survival in AML patients [178,179,180]. In rare cases, concomitant pathogen infections activating the immune system can lead spontaneously to remission in AML patients [181].

From these observations, immunotherapy, where therapeutic agents target immune cells rather than cancer cells, has been applied to AML. Graft-vs.-leukemia effect, namely the ability of donor immune cells to eradicate host leukemic cells after allo-HSCT, is a well-established proof-of-concept that an effective immune response could eliminate leukemia and represents the first example of immunotherapy. Nowadays, allo-HSCT is regarded as a potent immunotherapeutic treatment. However, the risk for severe and potentially fatal complications, including GVHD, must be considered [182].

The awareness of the complex and dynamic interactions between leukemic and immune cells potentially favoring blast proliferation and chemoresistance gave strength to the further development of immunotherapy protocol in AML [152]. A few years ago, the uncovering of the immune microenvironment biology had driven the development of immunotherapies based on the immune checkpoints inhibitors, including anti-CTLA-4, TIM-3, PD-1, and PD-L1 antibodies, adoptive T cell therapy, adoptive NK therapy, vaccines, monoclonal antibody therapy [183,184]. The available data on the immune-based therapeutic protocols have been reviewed elsewhere [185,186].

Several trials are ongoing to investigate the ideal immunotherapy setting [185]. In silico studies paved the way for identifying and correlating immunogenomic features with therapeutics/prognostic indicators in solid tumors, suggesting that the activation of immune pathways has a prognostic impact varying between different tumors [187]. Thus, also for AML, the study of tumor immunology acquired a central role in looking for an immune signature, able to predict which group of patients may benefit from personalized immunotherapies. A growing body of evidence indicates that disease-specific factors, including the immune milieu features, can strongly influence the response and the resistance to IC blockade. Functional T cell populations are required to guarantee the successful application of immunotherapies [123]. BM-mediated immune dysfunction and tumor immune evasion represent the main challenges for immunotherapy. Brück and collaborators described two main immunologic profiles in AML patients, differing from the age, T cell receptor clonality, and survival, suggesting a rationale for including the tumor immunology in risk stratification [152]. Notably, a correlation has been found between the immune landscape of AML and the clinical response [188]. In particular, by applying gene and protein profiling on primary AML BM samples, the identified IFN-dominant immune subtypes, obtained as the sum of IFN-γ signaling, inflammatory chemokine, and immune-related scores, predicts shorter overall survival, chemotherapy resistance, and the response of primary refractory/relapsed AML to flotetuzumab immunotherapy, which activates and expands residual T cells mediating AML cell eradication [188].

Similar to what was observed with conventional chemotherapy, primary and acquired resistance to immune therapy frequently occurs. Nowadays, it is becoming increasingly clear that both response and resistance to immune therapy are influenced by the tumor types and/or genotypes and the disease-specific immune milieu [189]. Indeed, besides the low neo-antigen burden of AML, one of the significant challenges for immunotherapeutic treatment of AML is overcoming the immunosuppressive mechanisms which characterize the disease. The combination of different immune-based therapies could represent a way [123]. For example, AML treatment with immunotherapeutic agents that activates T cells, leading to proinflammatory conditions, induces the PD-L1 upregulation in primary AML blasts preventing their lysis as an adaptive immune response–driven resistance mechanism [127]. AML patients non-responders to the treatment with azacitidine and nivolumab, an anti-PD-1, show the upregulation of CTLA-4 on CD4^+^ T cells, supporting the rationale to add ipilimumab, an anti-CTLA-4, to the regimen [190]. Thus, besides understanding the disease-specific immune microenvironment, the regulatory effect mediated by immune therapy on the immune system is crucial for designing more effective treatment.

## 5. Stromal/Immune BM Microenvironment Interplay

Besides the individual contribution of stromal and immune cells to induce and feed therapy resistance, extra layers of complexity are provided by their mutual interactions (Figure 3). It is well known that stromal cells can modulate immune cells’ proliferation, differentiation, and activity [191]. Specifically, MSCs hold a unique capacity to inhibit immune response as a cumulative result of different actions, among which are the inhibition of T-cell proliferation, blocking dendritic cell (DC) maturation, regulation of NK cells and macrophages, and above all, Treg induction [192]. MSCs are not immune-suppressive per se but acquire this capacity when stimulated by inflammatory signals [193]. MSC immune modulating capacity depends on several mediators among which, IDO1 plays a pivotal role. IDO1 exerts immune-regulating activity in different settings, including AML [194]. AML cells, but not normal HSCs, expressed IDO1 [195], which mediates immune tolerance [132] and correlates with a poor clinical outcome, as described above [142,143]. MSCs, including those isolated from myelodysplastic syndrome (MDS) and AML patients, up-regulate IDO1 following pro-inflammatory stimulation [86,193]. Specifically, after exposure to IFN-γ, IDO1-expressing MSCs become able to induce, in vitro and in vivo, fully functional Tregs recognized as essential contributors in microenvironment immunosuppression and ultimately in helping leukemic cells to evade immune surveillance, as mentioned before (Figure 3) [196,197,198]. Accordingly, an increased number of IDO1-expressing MSCs is associated with high levels of Tregs in AML patients [199]. In particular, co-culture with MSCs increases the immunosuppressive activity of Tregs [196]. Besides IDO1, the increase of inducible nitric oxide synthase in MSCs and the resulting production of nitric oxide (NO) play a major role in immunosuppression in murine systems [200]. A regulatory role of NO production in MSC-induced immune suppression has also been reported in hematological malignancies [201].

In the leukemic BM microenvironment, other inflammatory signals have been described. MSCs produce the inflammatory cytokine IL-6, which could have opposite effects on immune cells and immunosuppression [202]. Co-culture with leukemic cells upregulates TNF-α levels in MSCs [201]. Thus, MSC-produced TNF-α, a well-known crucial mediator in all steps of hematologic malignancies [203,204], may contribute to the generation of an inflammatory niche able to favor tumor growth and immune suppression. MSCs also produce IL-10, an anti-inflammatory cytokine, indicated as an adverse prognostic factor in AML patients [205], corroborating an emerging dual role of inflammation in the immune response [206]. In addition, MSCs secrete growth factors, such as GM-CSF and G-SCF, directly involved in drug resistance [26,207]. Thus, changes in the MSC-dependent inflammatory status of the microenvironment can collectively result in immune modulation and contribute to tumor progression and drug resistance within the BM niche. 

It is to be considered that chemotherapy by itself can induce inflammatory modifications in the leukemic BM microenvironment, including inflammatory cytokine release eventually affecting MSC immune-suppressive potential (Figure 3). Indeed, it has been demonstrated that chemotherapy-treated dying AML cells release ATP, which in turn up-regulates IDO1 expression in DCs, favors the induction of Tregs and contributes to creating an immune suppressive microenvironment in AML [208]. Accordingly, chemotherapy could potentially lead to the upregulation of IDO1 expression on MSCs, exacerbating the immune-tolerant microenvironment.

## 6. Conclusions

Many gaps remain to be filled, though it is becoming increasingly clear that, along with well-known cell-intrinsic determinants, the contribution of the BM microenvironment is crucial for a comprehensive understanding of AML. However, current leukemia treatments mainly focus on eradicating malignant cells and often neglect stromal and immune cell alterations.

Mutual interactions between the diverse components of the BM microenvironment, leukemic, stromal, and immune cells all participate in creating a permissive niche favorable to escape therapy and immune response. Thus, the direction of future research must be aimed at the design of synergistic therapies targeting multiple microenvironment-dependent mechanisms, thus turning out more effective control of AML progression and relapse.
